# Review of the Stepwise Laboratory Quality Improvement Process Towards Accreditation (SLIPTA) version 2:2015

**DOI:** 10.4102/ajlm.v9i1.1068

**Published:** 2020-10-28

**Authors:** Tjeerd A.M. Datema, Linda Oskam, Jacqueline E.W. Broerse, Paul R. Klatser

**Affiliations:** 1 DATOS B.V., Leiden, the Netherlands; 2 Department of Science Communication, Faculty of Science, Vrije Universiteit Amsterdam, Amsterdam, the Netherlands; 3 Athena Institute, Faculty of Science, Vrije Universiteit Amsterdam, Amsterdam, the Netherlands

**Keywords:** accreditation, ISO 15189, laboratory, SLIPTA, SLMTA, quality assurance, total quality management

## Abstract

**Background:**

In 2011 the Stepwise Laboratory Quality Improvement Process Towards Accreditation (SLIPTA) was launched, aimed at strengthening the quality and competence of African clinical, public health and reference laboratories. We reviewed the first version of the SLIPTA checklist in 2011. The continued development and publication of a new version of the International Organization for Standardization (ISO) 15189 standard demands a renewed review.

**Objective:**

This study aimed to determine the suitability of SLIPTA in guiding laboratories towards ISO 15189:2012 compliance and accreditation and provide recommendations for further SLIPTA improvement.

**Methods:**

The study was conducted between September 2018 and April 2019. Coverage of ISO 15189:2012 by SLIPTA checklist version 2:2015 was determined and the point distribution of the scoring system over the different sections of the SLIPTA checklist was re-investigated. These findings were compared with the review of the first version of the SLIPTA checklist (based on ISO 15189:2007) and with findings published on SLIPTA implementation and roll-out.

**Results:**

The coverage of ISO 15189 by the SLIPTA checklist has increased, even though ISO 15189:012 is more extensive than ISO 15189:2007. The point distribution is still skewed towards sections related to quality planning rather than quality control and improvement. Although to date 314 laboratories have been assessed, barriers for laboratories to participate in SLIPTA are high. Sustainability of SLIPTA results is insufficiently studied.

**Conclusion:**

SLIPTA checklist version 2:2015 has improved compared to earlier versions. We recommend increasing accessibility for laboratories to participate and increasing guidance for ISO-based quality management system implementation.

## Introduction

In 2011 the Stepwise Laboratory Quality Improvement Process Towards Accreditation (SLIPTA) was launched, aimed at strengthening quality and competence of clinical, public health and reference laboratories in the African region.^1,2,3,4^ The launch of SLIPTA was the result of a series of events. In 2008 the World Health Organization (WHO) and the United States Centers for Disease Control and Prevention (CDC) organised a conference on laboratory quality systems. One of the recommendations stated that laboratories in resource-limited settings should consider taking a staged approach towards implementation of a quality management system (QMS).^5,6^ In the following years, several resolutions on laboratory strengthening were drafted for the African region.^7,8^ In 2009, the WHO Regional Office for Africa (WHO-AFRO), in collaboration with CDC and other partners, launched the WHO-AFRO laboratory accreditation process and the Strengthening Laboratory Management Towards Accreditation (SLMTA) training and mentoring programme.^9,10,11,12^ In 2011, the WHO-AFRO accreditation process was renamed SLIPTA.^4,10,13^

In 2012, WHO-AFRO designated the African Society for Laboratory Medicine (ASLM) as the SLIPTA secretariat.^4,12,14^ The ASLM established SLIPTA’s implementation structure consisting of^4,14^:

- WHO-AFRO SLIPTA Focal Point, responsible for mobilisation of resources, providing guidance on content and implementation, and reviewing and updating SLIPTA.
- Ministry of Health SLIPTA Focal Point, responsible for -in-country promotion of laboratory improvement through SLIPTA. The ministry of health develops an implementation plan and prioritises SLIPTA applicant laboratories and allocates financial and human resources for SLIPTA implementation.
- ASLM-certified SLIPTA auditors, responsible for conducting audits and providing advice to auditees.
- SLIPTA independent advisory group, enrolling laboratories into the SLIPTA programme, organising audits and making a final decision on laboratory recognition through awarding a star rating based on audit reports (varying from zero to five stars) (Table 1). Star ratings are valid for a two-year period.^12,14^

**TABLE 1:** Stepwise Laboratory Quality Improvement Process Towards Accreditation checklist compliance levels versus star ratings.

Compliance level	Star rating
< 55%	No stars
55% – 64%	1 star
65% – 74%	2 stars
75% – 84%	3 stars
85% – 94%	4 stars
> 95%	5 stars

*Source:* World Health Organization Regional Office for Africa. Stepwise laboratory quailty improvement process towards accreditation (SLIPTA) checklist. Brazzaville; 2015

The ASLM aims to enrol 2500 laboratories in SLIPTA and have 250 public laboratories accredited to international standards by 2020.^12^

Not all laboratories can voluntarily participate in SLIPTA because ministries of health are encouraged to select laboratories in phases, considering tiered laboratory networks and giving precedence to laboratories that have already completed laboratory quality improvement training. Eligibility criteria include a SLIPTA self-audit score of 55% or higher, participation in proficiency testing schemes or alternative methods in the past 6 months and having conducted internal audits and a management review in the past 12 months. The laboratory should also have documented its QMS.^14^

Upon enrolment a laboratory is audited to determine its initial star rating. Laboratories are expected to work towards the next star. Laboratories that achieve a five-star rating are encouraged to apply for ISO 15189 accreditation.^14^

Key in the SLIPTA programme is the SLIPTA checklist, which is primarily based on ISO 15189 and, to a lesser extent, Clinical and Laboratory Standards Institute (CLSI) guideline QMS01-A4.^3,15^ Because laboratories with a five-star rating are encouraged to apply for accreditation, it is important to obtain insight into the coverage of ISO 15189 requirements by the SLIPTA checklist. This determines how much of the ISO 15189 requirements still must be addressed before full compliance with ISO 15189 is achieved. In 2011, the authors reviewed the first version of the SLIPTA checklist (published in 2009), referred to as the alpha version,^16^ and determined coverage of ISO 15189:2007.^1^ In that same year SLIPTA was revised and the SLIPTA alpha version became SLIPTA checklist v1.0.^11,12^ Because a new version of the ISO 15189 standard was published in 2012, SLIPTA checklist v2, based on ISO 15189:2012, was published in 2015.^3,12^ The current study determines coverage of ISO 15189:2012 by SLIPTA checklist v2:2015 and re-investigates the point division over the different sections of the SLIPTA checklist to determine the relative weight of QMS elements in SLIPTA. We also reviewed published SLIPTA implementation data.

This article informs potential users about SLIPTA’s suitability to guide laboratories towards ISO 15189:2012 compliance and accreditation and provides recommendations for further improvement of SLIPTA.

## Methods

### Ethical considerations

Ethical clearance was not required for this study.

### Study design

The study was conducted between September 2018 and April 2019. The methodology used in this study was adapted from Datema et al. 2011.^1^ The first analysis determined the SLIPTA checklist’s coverage of ISO 15189:2012 by linking each question of SLIPTA checklist v2:2015 to ISO 15189:2012 clauses. The second analysis provides insight into the point distribution of the scoring system over the different SLIPTA checklist sections. The SLIPTA checklist is divided into 12 sections corresponding with 12 QMS elements. For each section, points can be scored, the total of which determines the number of stars awarded. Excel 2016 (Microsoft, Redmond, Washington, United States) was used to analyse and compare the number of points that can be scored per section. Results were compared with results of the review of the SLIPTA checklist alpha version.^1^ In Datema et al. 2011, the 12 sections of the SLIPTA checklist were divided over the categories ‘Resource Management’, ‘Process Management’ and ‘Improvement Management’. In this article we renamed these categories to ‘quality planning’, ‘quality control’ and ‘quality improvement’, in line with the Juran Trilogy (Table 2).^17^ The overall intention of each SLIPTA checklist section led the categorisation process, which was identical to the review of the alpha version of the SLIPTA checklist.^1^ Hence, the overall aim of the sections assigned to the Juran category quality planning is to ensure quality of work before it is started, that is, before work can be conducted in a quality-assured way, proper organisation and functioning of equipment, purchasing and inventory management processes, good facilities and competent personnel are needed. As such, with the implementation of these elements the laboratory is ‘planning for quality’, justifying the decision for assigning these sections to the Juran category quality planning. Similarly, the primary, shared objective of the sections on process control, information management, documents and records, and client management is to control quality of work while it is being conducted. Therefore, these sections were assigned to the Juran category quality control. The sections on management reviews, evaluation and audits, occurrence or incident management and process improvement, and identification of non-conformities, corrective and preventive actions all share the common goal of continuously improving the quality of laboratory work. Therefore, these sections were assigned to the Juran category quality improvement.

**TABLE 2:** Distribution of Stepwise Laboratory Quality Improvement Process Towards Accreditation checklist v2:2015 sections over the different categories of the Juran Trilogy.

Category of the Juran Trilogy model	Related SLIPTA checklist v2:2015 sections	Related ISO 15189:2012 paragraphs
Quality planning	Facilities and biosafety	5.2
	Organization and personnel	4.1, 4.2, 5.1
	Equipment	5.3, 4.6
	Purchasing and inventory	5.3, 4.6
Quality control	Process control	5.4 – 5.7, 4.5
	Information management	5.8 – 5.10
	Documents and records	4.3, 4.13
	Client management & customer service	4.4, 4.7, 4.8
Quality improvement	Management reviews	4.15
	Evaluation and audits	4.14
	Occurrence/incident management & process improvement	4.12
	Identification of non-conformities, corrective and preventive actions	4.9 – 4.11

SLIPTA, Stepwise Laboratory Quality Improvement Process Towards Accreditation; ISO, International Organization for Standardization.

A PubMed search was conducted on 06 March 2019 to gather literature on outcomes of SLIPTA implementation and roll-out. The search terms were ‘SLIPTA’ OR ‘Stepwise Laboratory Improvement Process Towards Accreditation’ and the search yielded 29 hits. After primary and secondary selection based on title and abstract, and identification of additional reports through snowballing, a total of 23 articles were identified. Upon further scrutiny 12 papers were excluded because they did not report findings on SLIPTA implementation, roll-out, effectiveness or sustainability *per se.* Finally, 11 papers were included.

## Results

### Changes to Stepwise Laboratory Quality Improvement Process Towards Accreditation checklist v2:2015 compared with the alpha version

Structurally, SLIPTA checklist v2:2015 is very similar to the alpha version. The checklist is still divided over 12 sections based on the Quality System Essential structure developed by the CLSI.^15^ A notable change is the addition of one very detailed question on the presence and content of 36 specific standard operating procedures (SOPs).

#### ISO 15189:2012 coverage

The SLIPTA checklist v2:2015 addresses 82% of ISO 15189:2012 clauses, of which 35% are fully addressed, 47% addressed partially and 18% are not addressed at all. This is an improvement compared to the alpha version, which wholly or partially covered 52% of ISO 15189:2007 clauses. In some areas SLIPTA checklist v2:2015 is more detailed and prescriptive compared to ISO 15189 requirements, whereas in other areas ISO 15189 requirements are more extensive than the SLIPTA checklist.

#### Point distribution and relative weight (importance) of quality management system elements

**FIGURE 1:**
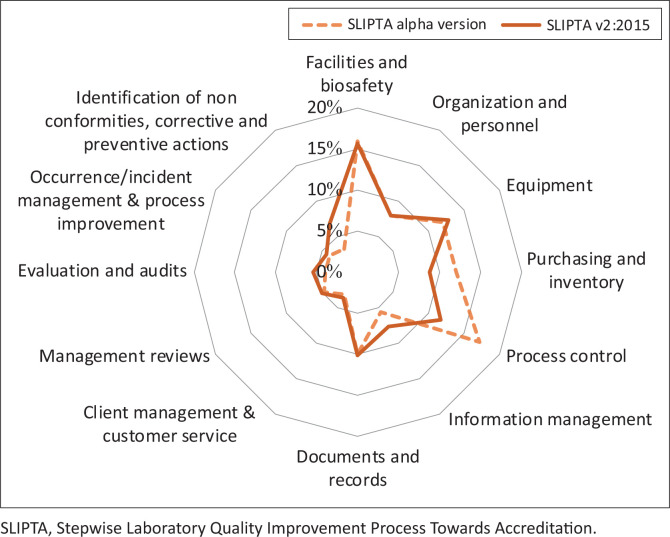
Relative point distribution of the scoring system over the different sections of Stepwise Laboratory Quality Improvement Process Towards Accreditation checklist alpha version and version 2:2015.

The total number of points in the SLIPTA checklist v2:2015 has increased from 250 to 275. In most sections a higher number of points can be scored compared to the alpha version. However, the relative weight of each section has remained similar due to the increase in the total number of points (see Figure 1).

**FIGURE 2:**
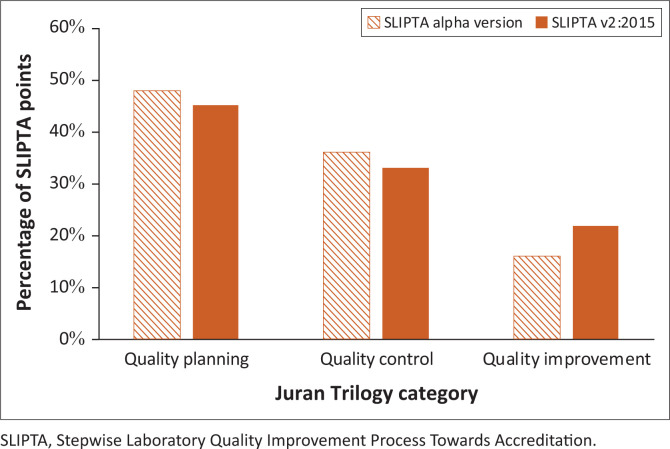
Relative point distribution of the scoring system of Stepwise Laboratory Quality Improvement Process Towards Accreditation checklist alpha version and version 2:2015 over the different categories of the Juran Trilogy.

When the point distribution of the SLIPTA checklist v2:2015 scoring system was analysed using the Juran Trilogy model, points were still heavily skewed towards quality planning (45% of the weight) and quality control (33%). Quality improvement received the lowest number of points (22%) (Figure 2).

### Outcomes of Stepwise Laboratory Quality Improvement Process Towards Accreditation roll-out

**FIGURE 3:**
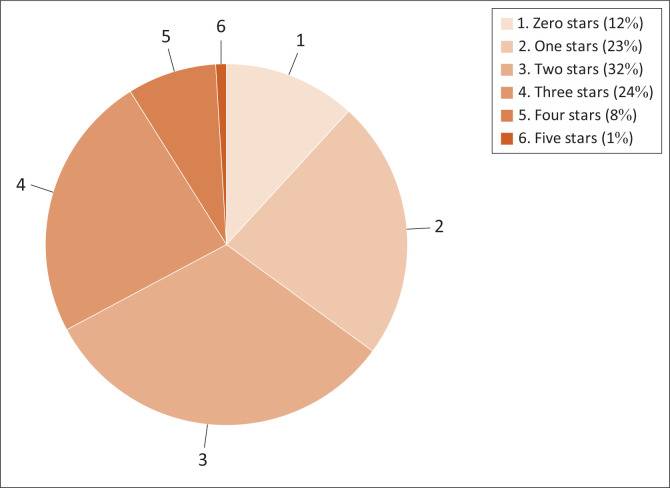
Percentage of laboratories per star rating, of a total of 314 laboratories, retrieved from Stepwise Laboratory Quality Improvement Process towards Accreditation database on 24 April 2019.^18^

Up to 24 April 2019, 314 laboratories had been audited in 20 countries, which is still far below the ambition to enrol 2500 laboratories by 2020 (Table 3).^18^ The percentage of laboratories per star rating is shown in Figure 3. The distribution is still in line with data published by Ndihokubwayo et al. in 2016, and Andiric et al. in 2018.^12,19^

**TABLE 3:** Number of laboratories per star level per country on 24 April 2019.^18^

Country	Number of laboratories						Total number of laboratories audited	
	0 stars	1 star	2 stars	3 stars	4 stars	5 stars	Absolute	Share of total (%)
Angola	2	1	2	0	0	0	5	2
Botswana	7	2	0	1	1	0	11	4
Burundi	0	2	1	1	1	0	5	2
Cameroon	0	3	6	6	1	0	16	5
eSwatini	0	1	4	2	0	0	7	2
Ethiopia	3	5	8	5	0	0	21	7
Ghana	1	2	3	4	5	0	15	5
Ivory Coast	3	4	2	0	0	0	9	3
Kenya	6	3	1	0	0	0	10	3
Lesotho	0	0	2	4	0	0	6	2
Malawi	0	1	7	3	1	0	12	4
Mozambique	0	9	8	2	0	0	19	6
Namibia	3	3	6	3	0	0	15	5
Nigeria	3	16	9	10	8	1	47	15
Rwanda	0	0	1	1	0	0	2	1
South Africa	0	2	8	7	4	2	23	7
Tanzania	4	9	13	12	0	0	38	12
Uganda	2	4	10	7	1	0	24	8
Zambia	0	5	2	0	0	0	7	2
Zimbabwe	3	1	8	7	3	0	22	7

**Total**	**37**	**73**	**101**	**75**	**25**	**3**	**314**	**100**

Although many papers have been published on the SLMTA training and mentoring programme, in which the SLIPTA checklist was used to measure progress, papers evaluating the SLIPTA initiative *per se* are scarce. The paper by Ndihokubwayo et al. (2016) is the only paper summarising SLIPTA implementation and lessons learned.^12^ Two additional papers were found that present SLIPTA implementation findings, but these also combined SLIPTA with additional assistance.^20,21^ Both studies indicated that combining SLIPTA with mentoring has a positive effect on QMS implementation, although neither control laboratories nor findings on sustainability of this model were included.^20,21^

Ndihokubwayo et al. found that SLIPTA laboratories performed most poorly on the internal audit and corrective action sections.^12^ This finding is corroborated by other studies.^22,23,24,25,26,27,28^ They further state that advocacy for laboratory strengthening is key to the SLIPTA process (as it is owned by the ministry of health) and that particularly Francophone and Lusophone countries are not well covered. An explanation for the latter finding might be that SLIPTA was initially implemented through the United States President’s Emergency Plan for AIDS Relief, which is oriented towards Anglophone countries.^12^

## Discussion

SLIPTA checklist v2:2015 has improved compared to the alpha version. Even though ISO 15189:2012 is more extensive than ISO 15189:2007, ISO 15189 coverage has increased, decreasing the gap that still needs to be bridged by 5-star laboratories aiming for ISO 15189 accreditation. However, the gap is still considerable: only 35% of ISO 15189 clauses are fully covered, leaving 47% partially covered and 18% not covered at all. Compliance with some of these requirements may be reached as part of the continuous improvement process which 5-star laboratories may have already partially implemented.

The main point for SLIPTA improvement remains the absence of prioritisation of QMS implementation activities. There are no conditions for the different star ratings (other than the star rating thresholds) that encourage laboratories to implement the QMS in a specific, rational manner, indicating that QMS implementation guidance remains limited. Most SLIPTA checklist v2:2015 questions are supplemented with short notes including examples, but neither a stepwise plan nor a detailed explanation of implementation of requirements is provided, showing that SLIPTA remains primarily an assessment checklist. SLIPTA auditors may provide advice on implementation during assessments^4,12^ but this might come late, for laboratories may first try to implement a QMS before being assessed as laboratories must score at least 55% in self-assessment to meet eligibility criteria for enrolment.^14^

Currently, most points can be scored in SLIPTA checklist sections related to quality planning, followed by quality control. The least number of points can be scored on quality improvement. This creates an imbalance. Implementing a QMS is a ‘systems approach’: all QMS elements work together to create a sustainable system that can deliver quality-assured laboratory services and continuously improve itself. When one QMS element is not (correctly) functioning, quality assurance of overall laboratory services and continuous quality improvement may be compromised. Therefore, one could argue that SLIPTA should award an equal number of points for each section. On the other hand, the skewed point distribution may point laboratories in the right direction by encouraging them to address sections related to quality planning first because of the high number of points that can be scored in this category, as was also argued by Datema et al. in 2011 (although no evidence was found in literature supporting this hypothesis).^1^ However, this is counterbalanced by the higher amount of work required for implementing the sections related to quality planning.

Another improvement opportunity for the SLIPTA checklist is the imbalance in the effort required to earn points per question. For example, for question 1.5 one needs to develop 36 SOPs to earn five points, whereas by developing a list of documents used in the laboratory (question 1.4), making sure that SOPs are accessible to staff (question 1.6), and indicating date of authorisation, location and date of discontinuation on each SOP (question 1.8) one can score a total of six points. Writing 36 SOPs obviously requires considerably more effort.

### Stepwise Laboratory Quality Improvement Process Towards Accreditation implementation and roll-out

A strong point is that SLIPTA requires the ministry of health to play an active role; government commitment has been shown to be key to success in both SLIPTA^21^ and SLMTA.^11,12,20,24,25,27,29,30,31,32,33,34,35^ Also, SLIPTA can be well combined with other guidance methods for laboratory accreditation as is evident from many studies on SLMTA implementation.^20,22,27,29,30,33,34,35,36^

A major downside is the indirect accessibility of the SLIPTA programme: laboratories are selected by the ministry of health for participation. Only 27 of the 47 WHO-AFRO member states have established a SLIPTA focal point within their ministry of health. Moreover, the programme has a language bias towards Anglophone countries.^12,18,28^ Also, up to April 2019, 314 laboratories had been assessed, which is low considering that Kampala, the capital of Uganda, alone counts 954 laboratories^18,37^ and that ASLM aimed to include 2500 laboratories by 2020.^12^

### Recommendations for improvement

The level of guidance provided by SLIPTA for QMS implementation could be increased by making better use of the star rating system. Currently, stars are simply awarded based on the number of points scored, regardless of the section these points are scored in. Setting certain benchmarks and conditions for the different star ratings may improve guidance. An example could be the definition of key questions that have to be implemented for each star rating. This may help laboratories in using a rational approach towards QMS implementation. It may also assist laboratories in lower tiers of laboratory networks, for which ISO 15189:2012 is not (yet) feasible, to implement a basic yet functional QMS. The phased approach incorporated in the WHO Laboratory Quality Stepwise Implementation (LQSI) tool as well as the tiered approach of the Laboratory Quality Management System Stepwise Improvement Process (LQMS-SIP) used in the Caribbean region could serve as models in assigning key questions to the different star ratings, which would also contribute to harmonisation of SLIPTA with these laboratory strengthening tools and initiatives.^38,39,40^

Sustainability is a challenge for laboratory strengthening efforts. Literature on SLIPTA implementation does not provide sufficient clarity on sustainability. SLIPTA assessments, like accreditation assessments in general, are snapshot measurements of laboratory compliance, creating the risk that the efforts may weaken after an assessment, as was also witnessed in SLMTA evaluations.^11,24,32^ A possible measure to decrease this risk is announcing assessment visits only shortly before they are scheduled, leaving just enough time for a laboratory to prepare logistics but not for correction of elements that would otherwise not have been corrected, yielding a more representative view of the daily practice. Another measure is the adoption of a point scoring system that awards negative points for questions that are not in place anymore compared to the previous assessment. This emphasises the importance of quality *assurance* and may be an extra driver for the laboratory to ensure continued compliance. In the case of SLIPTA, both measures could be adopted without requiring major revisions as it would primarily require amendment of the audit scoring section of the SLIPTA checklist. It should be noted that SLIPTA checklist v2:2015 already indicates that audit scores should be based on laboratory performance during the 12 months preceding the SLIPTA audit, which is an encouragement for laboratories to maintain compliance.^3^

The last recommendations relate to implementation and roll-out of SLIPTA: increasing the accessibility of SLIPTA by increasing the capacity, among others through ensuring the presence of SLIPTA focal points at each ministry of health and training of more SLIPTA auditors. This should include training of more SLIPTA auditors fluent in Portuguese and French to increase accessibility for laboratories in Lusophone and Francophone countries.

### Limitations

The analysis using the Juran Trilogy model was limited to categorisation of overall SLIPTA sections. Although the authors are aware that the same analysis can be conducted at the individual question level, the decision was made to categorise based on the overall intention of each SLIPTA section and, therefore, categorise each SLIPTA checklist section as a whole as described in the methods section. This was also required to enable comparison with the review of the alpha version of the checklist. This study is a desk-based review. Ideally, the findings of this study should be triangulated through an observational study monitoring SLIPTA implementation with a sufficiently large sample size. This would enable substantiation of the findings and may reveal additional opportunities for improvement of the SLIPTA checklist and programme.

### Conclusion

SLIPTA checklist v2:2015 has improved compared to the alpha version. Suggestions for improvement are mainly related to the point scoring system, including the designation of key questions to specific star ratings to improve the level of guidance for implementation of a QMS. The LQSI tool and LQMS-SIP could serve as examples, leading to harmonisation of SLIPTA with these tools. Recommendations for enhanced SLIPTA roll-out include increasing accessibility by translation into French and Portuguese, training more auditors, and increasing capacity for participation.
